# A case–control study of the association between self-reported occupational and recreational physical activity and lung cancer

**DOI:** 10.1097/MD.0000000000007923

**Published:** 2017-09-08

**Authors:** Fei He, Li-mei Chen, Wei-min Xiong, Qiu-ping Xu, Ren-dong Xiao, Xu Li, Tao Lin, Lin Cai

**Affiliations:** a Department of Epidemiology, School of Public Health; b Key Laboratory of Ministry of Education for Gastrointestinal Cancer; c Fujian Provincial Key Laboratory of Environment factors and Cancer, School of Public Health; d Department of Sports, School of Basic Medicine, Fujian Medical University; e Department of Thoracic Surgery, The First Affiliated Hospital of Fujian Medical University, Fuzhou, China.

**Keywords:** body mass index, chronic lung disease, lung cancer, physical activity, smoking

## Abstract

This case–control study with a Fujian population investigated whether self-reported occupational and recreational physical activity may be associated with lung cancer.

The population comprised 1622 patients with newly diagnosed primary lung cancer and 1622 age- and gender-matched healthy controls.

High-intensity occupational physical activity was associated with significantly higher risk of lung cancer (OR = 1.354, 95% CI: 1.068–1.717), especially nonsmall cell lung carcinoma (OR = 1.384, 95% CI: 1.087–1.762). Moderate or low intensity recreational physical activity was associated with reduced risk of lung cancer. The protective effect of recreational physical activity was observed in current or former smokers, but not never-smokers, and in subjects with normal or high BMI, but not low BMI, as well as people without a history of chronic lung disease. The frequency of recreational physical activity was associated with a linear reduction in the risk of lung cancer (*P* < .001), and also specifically nonsmall cell lung cancer (*P* < .001).

Occupational and recreational physical activity was associated with different effects on the risk of lung cancer in a Fujian population. While recreational physical activity was associated with decreased risk of lung cancer, occupational physical activity was associated with increased risk of lung cancer.

## 1 Introduction

Lung cancer is one of the most common and serious types of cancer in the world. According to the GLOBOCAN 2012 report^[1]^ released by the International Agency for Research on Cancer, age-standardized morbidity and mortality rates for lung cancer were 34.2 per 100,000 and 30.0 per 100,000 respectively in men, and 13.6 per 100,000 and 11.1 per 100,000 respectively in women. Lung cancer accounts for the largest proportion (13.0%) of newly diagnosed cancer cases in the world, and highest proportion of cancer death (19.4%).^[1]^ In China, the age standardized incidence rate of lung cancer among men is 48.4/100,000, making it the most common cancer in men. Among women, lung cancer incidence ranks second only after breast cancer, with an age standardized incidence rate of 21.9/100,000. Regardless of gender, lung cancer is ranked the leading cause of death among malignant tumors.^[2,3]^

Smoking tobacco is the single most important risk factor for lung cancer.^[4]^ The risk of developing lung cancer is 14 times higher in smokers compared with nonsmokers.^[5]^ However, not all smokers suffer from lung cancer, suggesting that the etiology of lung cancer is multifactorial. Accumulating evidence suggests that lack of exercise may also be an important risk factor for cancer.^[6]^ Regular physical activity can improve lung function and reduce tobacco-induced lung and airway injury.^[7]^ Most studies on the subject have considered that smoking is a confounding factor. The results of a recent review^[8]^ suggested that the protective effect of physical activity is only found in current and former smokers, but not never-smokers. Other factors such as obesity and chronic lung disease are also possible confounding factors that may obscure an association between physical activity and lung cancer.

The association between physical activity and the risk of lung cancer is complex, and a recent study suggested that not all physical activity has the same effect.^[9]^ Most previous studies have focused on the role of recreational (as opposed to occupational) physical activity. A systemic review and meta-analysis of 28 relevant studies concluded that regular recreational physical activity may reduce lung cancer risk.^[10]^

Studies on the effect of occupational physical activity have been less common. Some studies have reported that occupational physical activity may increase the risk of lung cancer in men,^[9]^ and that men with standing occupations had higher risk of lung cancer than did men with sitting occupations.^[9,11]^ However, other studies showed that occupational physical activity does not affect lung cancer risk.^[12,13]^

The present case–control study explored an association between self-reported occupational and recreational physical activity and lung cancer in a Fujian population.

## 2 Methods

### 2.1 Study subjects

The Ethics Committee of Fujian Medical University approved the study and all the subjects signed the consent form. The present study applied a hospital-based on-going case–control design.

From January 2006 to June 2015, we enrolled cases of newly diagnosed primary lung cancer who were admitted to First Affiliated Hospital of Fujian Medical University, Fujian Medical University Affiliated Union Hospital, or Fuzhou General Hospital of Nanjing Military Region. The diagnoses were made by clinical examination and bronchoscopy. Control subjects were selected by frequency matching according to age (±2 years) and gender, among the healthy visitors to nononcology departments during the same period and healthy people in the community. All the subjects had lived in Fujian province in China for more than 10 years and were willing to cooperate with the survey and able to answer the questions clearly.

### 2.2 Survey content and variable definition

The survey participants were interviewed by uniformly trained investigators according to a structured questionnaire.^[14,15]^ The interview included questions concerning the following broad areas: baseline characteristics; smoking status; exposure to environmental tobacco smoke (ETS); physical activity; history of chronic lung disease; family history of lung cancer; and alcohol and tea consumption.

Body mass index (BMI) was calculated as body weight (kg)/height^2^ (m^2^), and categorized as low (<18.5), normal (18.5 ≤ BMI < 25.0), and overweight or obese (≥25.00). A smoker was defined as someone who had cumulatively smoked >100 cigarettes. A former smoker was a person who previously smoked, but had not smoked for more than 3 consecutive months. Exposure to ETS was defined as never directly smoked, but inhales the smoke produced by cigarettes or exhaled by smokers >15 minutes per day. Drinking alcohol was considered as ≥1 time/week for more than 6 months, and drinking tea as ≥1 cup/week for more than 6 months.

Physical activity during the past 2 years was quantified. Occupational physical activity was rated as low, moderate, or high intensity, in accordance with the Reference Standard of Labor Intensity recommended by the Chinese Nutrition Society in the year 2000.^[16]^ Specifically, low intensity refers to activities performed by office workers; repairers of electrical appliances, watches, and clocks; sales staff; hotel attendants; chemical experiment operators; and lecturers. Moderate intensity occupations include those in which participants sit or stand for 25% of the work time, while 75% is spent on moderate intensity special vocational activities such as student daily activities; motor vehicle driving; electrical installation; lathe operation; and metalwork cutting. High intensity occupations are those in which 40% of the time is spent sitting or standing, while 60% is spent on high intensity activities such as nonmechanized agricultural labor, steel, dance, sports, loading and unloading, and mining.

The intensity of recreational physical activity or exercise was classified as moderate (sweat inducing) or low (nonsweat-inducing, such as walking). Recreational physical activity was also rated as occasional (2–3×/week) or frequent (>3×/week).

### 2.3 Statistical analysis

All statistical analyzes in this study were performed using SPSS 23.0 statistical software. The Chi-squared test and *t* test were used to compare the baseline demographic characteristics of the case and control groups. An unconditional logistic regression model was used to analyze the correlation between physical activity and lung cancer. Odds ratios (ORs) and 95% confidence intervals (CIs) were calculated. Associations between physical activity and lung cancer were analyzed with logistic regression analysis based on smoking status, ETS exposure, history of chronic lung diseases, and BMI. Multiple logistic regression analysis was performed using the Backward Stepwise Wald method to identify the risk factors for lung cancer. The *P*-value of the results was 2-tailed, with a test level alpha (α) = 0.05.

## 3 Results

### 3.1 Baseline characteristics

The study population comprised 3244 individuals, including 1622 patients with lung cancer and 1622 age- and gender-matched healthy controls. The baseline characteristics are shown in Table 1. The age, gender, ethnicity, and marital status of the 2 groups were well-matched, but statistically significant differences were found with regard to BMI, education level, occupation, family history of lung cancer, history of chronic lung disease, smoking status, exposure to ETS, fruit, fish, alcohol and tea consumption (Table 1).

**Table 1:**
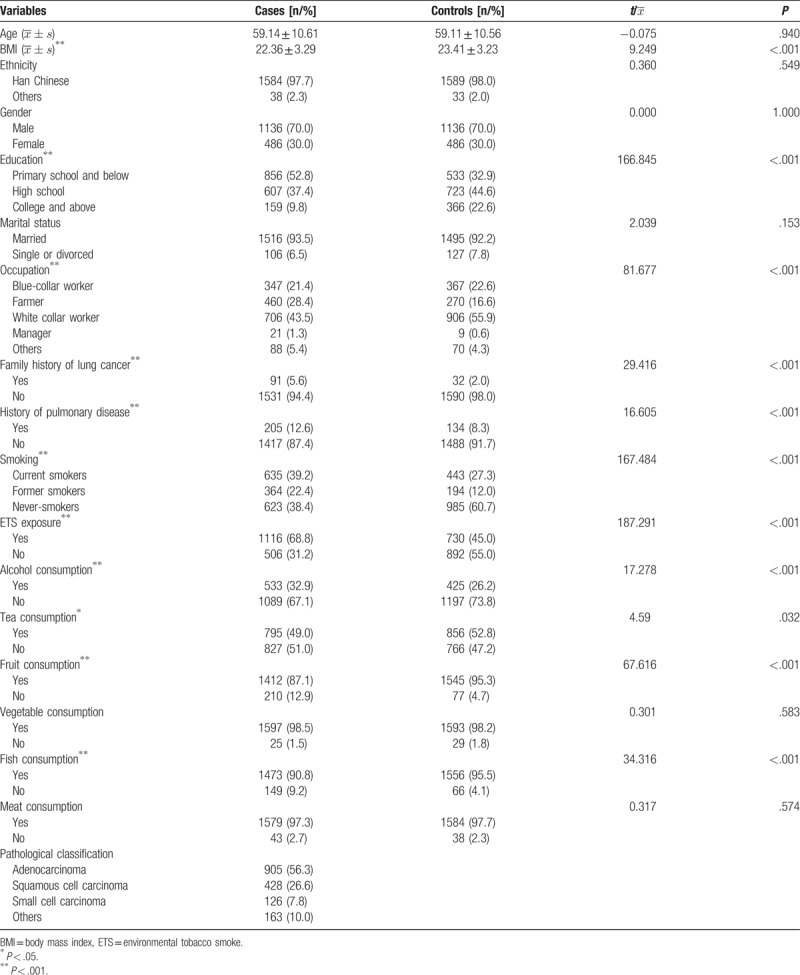
The baseline characteristics of the study population.

### 3.2 Association between physical activity and pathological types of lung cancer

The risk of any lung cancer was significantly higher (OR: 1.354, 95% CI: 1.068–1.717) in subjects who engaged in high intensity occupational physical activity compared to low intensity. Recreational physical activity at both moderate (frequent: OR = 0.708, 95% CI: 0.578–0.867; occasional: OR = 0.666, 95% CI: 0.535–0.830) and low intensity (frequent: OR = 0.623, 95% CI: 0.519–0.748; occasional: OR = 0.704, 95% CI: 0.565–0.876) was associated with a lower risk of any lung cancer (Table 2). A linear correlation was found between the frequency of recreational physical activity, regardless of intensity, and risk of any lung cancer (*P* < .001), that is, the more frequent the physical activity, the higher the risk reduction. A similar linear correlation (*P* < .001) was also observed when only nonsmall cell lung cancers were included. The risk of nonsmall cell lung cancer in those exposed to high intensity occupational activity was 1.384-fold (95% CI: 1.087–1.762) that of those only exposed to low intensity (Table 2).

**Table 2:**
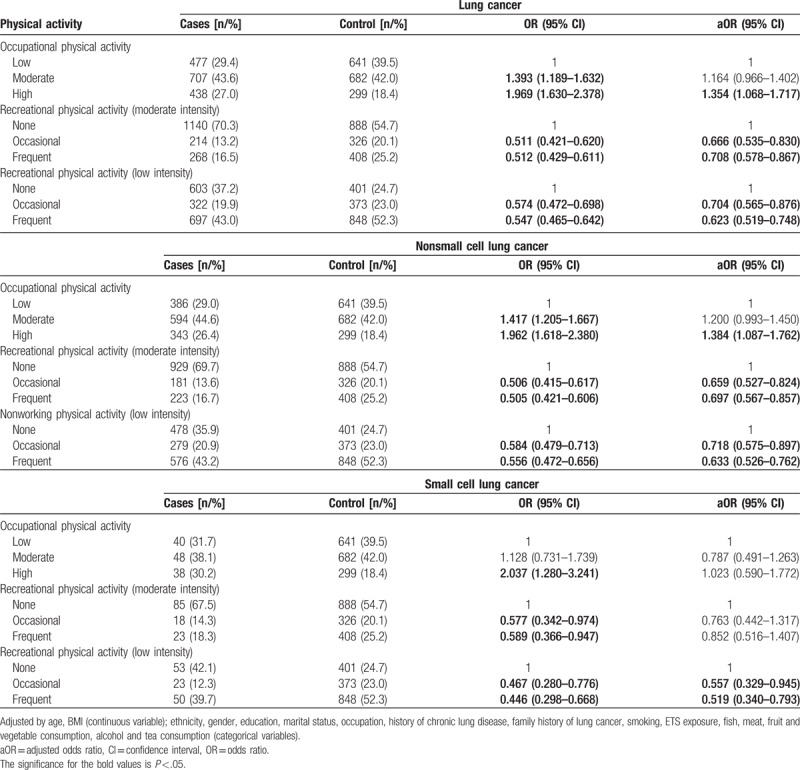
Association between different types of physical activity and lung cancer.

### 3.3 Associations between lung cancer and physical activity of people according to BMI

After adjustment for other confounding factors, physical activity did not affect risk of lung cancer in subjects with low BMI. In subjects with normal, both moderate and low intensity recreational physical activity was associated with lower risk of lung cancer, both when performed occasionally (moderate intensity: OR = 0.591, 95% CI: 0.453–0.771; low intensity: OR = 0.744, 95% CI: 0.582–0.950) and frequently (moderate intensity: OR = 0.744, 95% CI: 0.582–0.950; low intensity: OR = 0.660, 95% CI: 0.529–0.823). In overweight and obese subjects, low intensity recreational physical activity was associated with lower risk of lung cancer (frequent: OR = 0.518, 95% CI: 0.355–0.758; occasional: OR = 0.577, 95% CI: 0.369–0.903; Table 3).

**Table 3:**
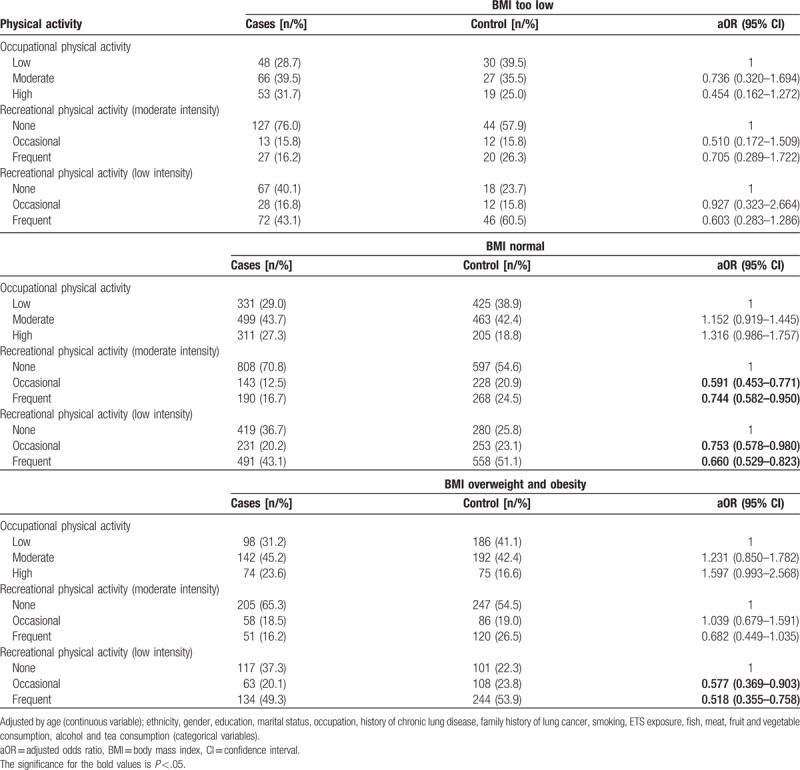
Association between physical activity and lung cancer by BMI.

### 3.4 Associations between lung cancer and physical activity in people according to smoking and ETS exposure status

After adjustment for other factors, in both the current and former smokers, recreational physical activity was associated with reduced risk of lung cancer. In current smokers, moderate intensity recreational physical activity was associated with 29.8% (95% CI: 0.495–0.997) lower risk when performed frequently and 60.1% (95% CI: 0.268–0.596) when performed occasionally. Low intensity recreational physical activity was associated with 48.1% (95% CI: 0.380–0.708) lower risk when performed frequently and 47.9% (95% CI: 0.381–0.712) when performed occasionally.

In former smokers, frequent moderate-intensity recreational physical activity was associated with lower 39.6% (95% CI: 0.366–0.998) lower risk of lung cancer. Frequent low-intensity physical activity was associated with 52.6% (95% CI: 0.296–0.761) lower risk, while occasional low-intensity physical activity was associated with 49.3% (95% CI: 0.282–0.911) lower risk. Physical activity was not associated with altered risk for lung cancer in never-smokers (Table 4).

**Table 4:**
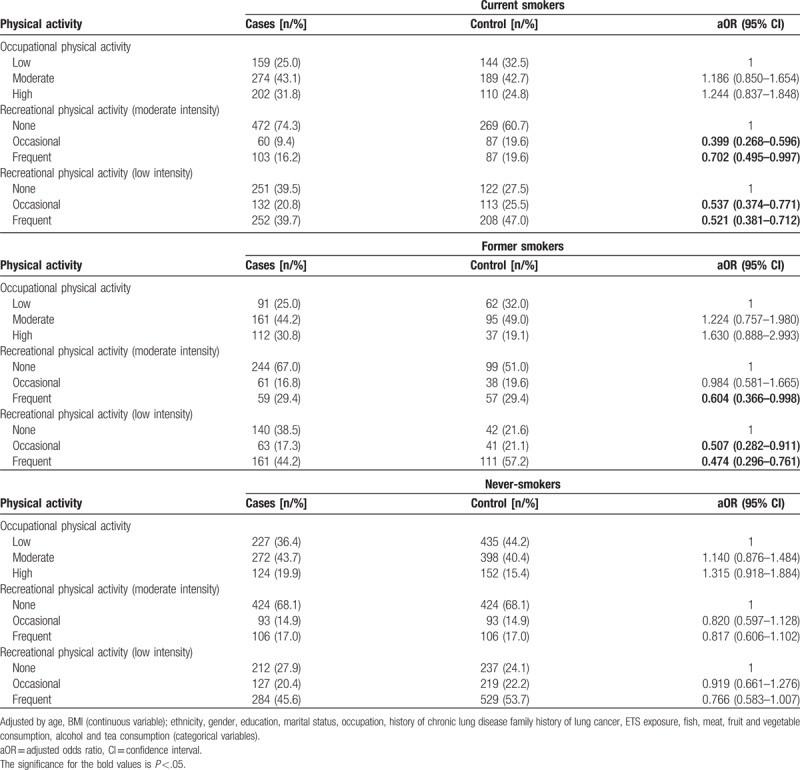
Association between physical activity and lung cancer according to smoking history.

Recreational physical activity was also associated with lower lung cancer risk in both ETS-exposed and not ETS-exposed subjects. In subjects not exposed to ETS, the OR was 0.542 (95% CI: 0.391–0.751) for frequent moderate intensity recreational physical activity and 0.693 (95% CI: 0.494–0.974) for occasional moderate intensity recreational physical activity. Low intensity recreational physical activity was also associated with lower lung cancer risk when performed frequently (OR 0.550, 95% CI: 0.414–0.731). However, in ETS-exposed subjects, low-intensity recreational physical activity was only associated with 30.3% (95% CI: 0.547–0.890) lower risk when conducted frequently, and 29.4% (95% CI: 0.530–0.941) when conducted occasionally (Table 5).

**Table 5:**
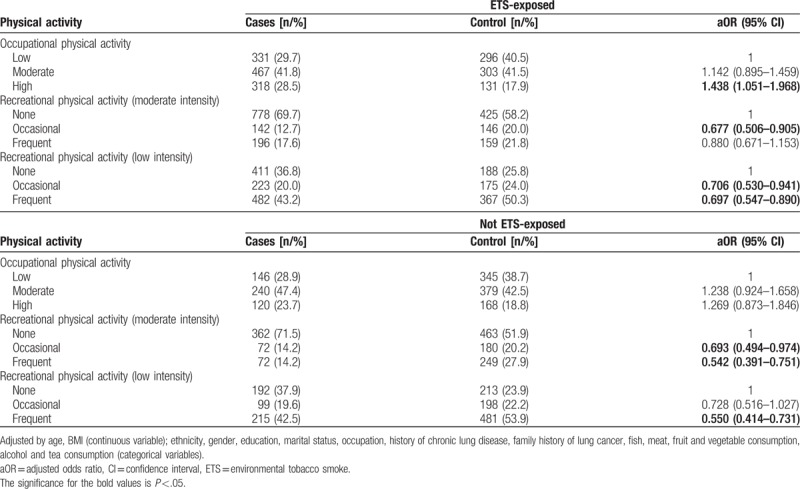
Association between physical activity and lung cancer according to exposure to ETS.

### 3.5 Associations between lung cancer and physical activity according to chronic lung disease history

After adjustment for other confounding factors, subjects who suffer from a chronic disease only had a reduced risk of lung cancer with frequent low intensity recreational physical activity (OR: 0.498, 95% CI: 0.254–0.975). Subjects who had never suffered from a chronic lung disease had a reduced risk of lung cancer when involved in moderate intensity recreational physical activity. Frequently performing moderate intensity recreational physical activity (OR = 0.672, 95% CI: 0.542–0.834) and occasionally performing such (OR = 0.643, 95% CI: 0.508–0.814) was associated with lower lung cancer risks (Table 6).

**Table 6:**
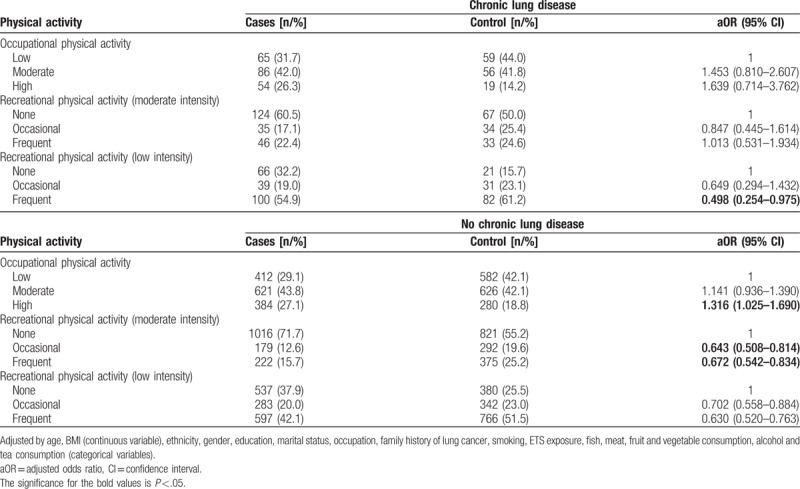
Association between physical activity and lung cancer according to history of lung disease.

### 3.6 Risk factors for lung cancer identified by multiple logistic regression analysis

Multiple logistic regression using the Backward Stepwise Wald method identifies gender, education, marital status, occupation, family history of lung cancer, history of chronic lung disease, current smoking, former smoking, ETS exposure, BMI, tea, fish and fruit consumptions are factors that can affect the risk for lung cancer. High intensity occupational physical activity (*P* = .021), low intensity (*P* = .012) and moderate intensity recreational physical activity (*P* = .005) have all been shown to affect lung cancer risk (Table 7). These variables are all adjusted for in the calculation of OR in the above analysis.

**Table 7:**
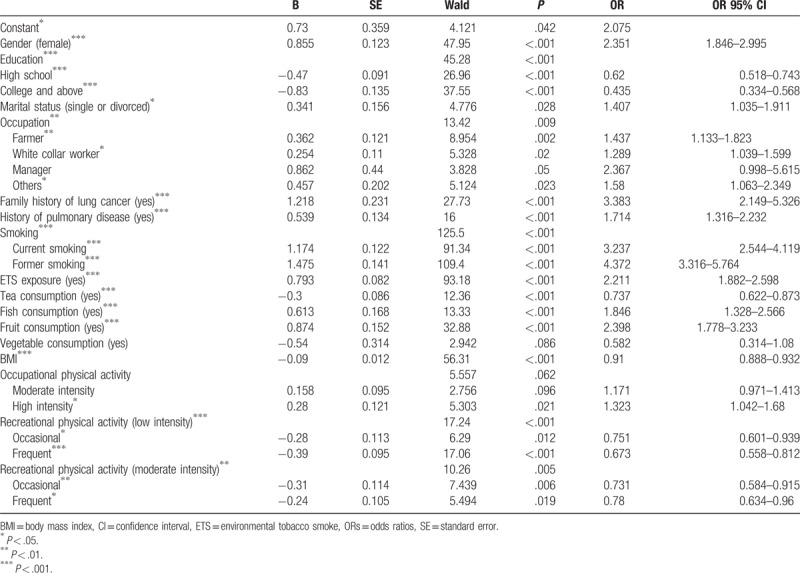
Multiple logistic regression analysis of the risk factors for lung cancer.

## 4 Discussion

Physical activity refers to activity that is produced by skeletal muscle contraction, resulting in energy expenditure, and is associated with daily life, work, and recreational activities, including occupational physical activity, commuting to and from work, housework, shopping, sports, and exercise. Recreational physical activity is characterized by short-term, intensive energy consumption, whereas occupational physical activity is characterized by a lower energy consumption rate over a longer period of time.^[17]^ The present study showed that occupational and recreational physical activity was associated with different effects on lung cancer risk.

Physical activity is a diverse and complex concept. Several methods for quantification and categorization of physical activity can be found in the literature.^[10]^ The World Health Organization recommends ≥150 minutes of moderate physical activity per week or ≥75 minutes of high intensity physical activity per week. Moderate intensity physical activity is defined as activity that noticeably increases the heart rate such brisk walking and dancing, while high intensity physical activity is defined as activity that causes rapid breathing and substantial increase in heart rate such as running and fast cycling.^[18]^

The intensity of activity can also be measured according to the energy metabolic equivalent (MET) of the participant. The ratio of metabolic rate during exercise to the standard resting metabolic rate (4.184 KJ kg^–1^ h^–1^) represents the intensity of a particular physical activity, and according to the activity type and intensity, different physical activity has a specific MET value. In the present study, information about the activity intensity and activity frequency were acquired through questionnaires or interviewing the subjects, and asking the subject to define their own intensity level, generally defined as moderate or low. Moderate intensity refers to physical activity or physical exercise that reaches the degree of sweating, and low intensity to nonsweat-inducing exercise such as walking. The subjects were also asked to state their physical activity frequency in terms of the number of times physical activity was performed (times/week) during the last 2 years. Occasional was defined at recreational physical activity 2 to 3 times per week, and frequent as more than 3 times per week. The intensity of occupational physical activity was defined according to the Reference Standard of Labor Intensity recommended by the Chinese Nutrition Society in 2000.^[19]^

Through a review of the literature, 8 case–control and 29 cohort studies were found, as well as studies with other designs that explored associations between physical activity and lung cancer. Eighteen of the studies^[20–36]^ found that physical activity and lung cancer was negatively correlated. Nineteen studies^[8,11–13,34,37–49]^ reported no association between physical activity and lung cancer. Eight studies looked specifically at occupational physical activity and lung cancer.^[11,13,21,34,37,38,48,50]^ Only Brownson et al^[21]^ reported that lung cancer had nothing to do with occupational physical activity. The present study found that high intensity occupational physical activity was associated with increased risk of lung cancer, especially nonsmall cell lung cancer. On the other hand, recreational physical activity was associated with reduced risk of developing lung cancer, especially nonsmall cell lung cancer, in former and past smokers, non-ETS-exposed subjects, and subjects without a history of chronic lung diseases.

In the present study, the control group contained a higher percentage of never-smokers compared to the case group (Table 1). How smoking status affects the association between physical activity and lung cancer was assessed in a meta-analysis,^[8]^ which compared 25 observational studies. The results suggested that physical activity negatively correlated with lung cancer risk in former (RR = 0.68, 95% CI = 0.51–0.90) and current smokers (RR = 0.80, 95% CI = 0.70–0.90), but had no association with lung cancer risk of nonsmokers (RR = 1.05, 95% CI = 0.78–1.40, *P*_interaction_ = .26). Adjusting for the level of smoking did not change these results (*P*_interaction_ = .73). This is highly consistent with the results of the present study. In addition, this study further found that exposure to ETS did not affect the association between physical activity and lung cancer (*P*_interaction_ = .16). However, in the present study, high intensity occupational physical activity increased the risk of lung cancer in subjects exposed to ETS, but does not affect subjects that are not ETS-exposed. Recreational physical activity reduces lung cancer risk in both ETS-exposed and not ETS-exposed (Table 5).

The present study has a retrospective case–control design. Berkin bias was minimized by including subjects from four different hospitals in the Fujian province: First Affiliated Hospital of Fujian Medical University, Fujian Medical University Affiliated Union Hospital and Fuzhou General Hospital of Nanjing Military Region, and also including healthy controls subjects from the community. To eliminate potential confounding factors and objectively assess the association between physical activity and lung cancer, factors such as obesity, chronic lung disease, intake of fruit/vegetables/meat/fish and other factors must therefore be considered in addition to adjusting for smoking and ETS exposure. Air pollution is also a known risk factor for lung cancer.^[51]^ However, since all included subjects have lived in the Fujian province in China for more than 10 years, the exposure to air pollution was assumed to be similar among the subjects. In the present study, the control group had a higher mean BMI than did the lung cancer group. There is a difference in the level of physical activity for people with different BMI levels. One study^[52]^ showed that people with higher BMIs participated in more recreational physical activity, but they infrequently chose walking or cycling for commuting. A history of chronic lung disease is related to lung cancer risk, and disease itself can cause a lack of physical activity in patients.^[52]^ Thus, these factors’ influence needed to be considered when studying the association between lung cancer and physical activity. Other factors that could potentially influence the validity of the conclusions drawn from the present study includes recall and selection biases. A prospective cohort study would be valuable to further confirm the effect of physical activity on lung cancer.

The present study found that low-intensity physical activity had a protective effect on subjects with BMI in the normal or overweight/obese range, but no effect in subjects with low BMI (Table 3). These results are in contrast to that of Mao et al^[30]^ in which physical activity was shown to significantly reduce the risk of lung cancer in people with low BMI, while no relation was found in people with high BMI. This may be caused by inconsistency in the stratification of BMI. Mao et al^[30]^ stratified BMI as <25, 25–30, and ≥30 kg/m^2^, while the present study stratified BMI according to the criteria of the World Health Organization for adults (<18.5, 18.5–24.9, ≥25 kg/m^2^). To the authors’ best knowledge, no previous study has included history of chronic lung disease as an adjustment factor or to stratify their analysis. The present study found that moderate intensity recreational physical activity negatively correlated and high intensity occupational physical activity positively correlated with lung cancer risk only in people without a history of chronic lung disease.

Several hypotheses may potentially explain a biological mechanism underlying a reduction in lung cancer risk through recreational physical activity. For example, exercise can affect growth factors such as insulin-like growth factor (IGF) and IGF binding protein (IGFBP), thereby altering tumor progression. High levels of circulating IGF-1 may increase lung cancer risk, while high levels of IGFBP-3 may reduce the risk of lung cancer.^[30]^

In addition, physical activity can significantly reduce insulin, glucose, and triglyceride levels, and increase the level of high-density lipoprotein cholesterol, which may be associated with reduction in lung cancer risk.^[53]^ Another possibility is that physical activity may enhance immune system function^[12,38]^ by increasing the number and activity of macrophages, natural killer cells, lymphokine-activated killer cells, and lymphokine regulated cytokines, as well as increasing mitogen-induced lymphocyte proliferation rates. Smoking and ETS exposure can lead to respiratory inflammation and increase oxidative stress. Physical activity can increase the secretion of endogenous free radical scavengers which decrease oxidative stress and inflammation, thereby increasing lung ventilation and perfusion to reduce the risk of lung cancer.^[54–56]^

## 5 Conclusion

In conclusion, we found a significant association between the type and intensity of physical activity and lung cancer in this Fujian population. Occupational and recreational physical activity was associated with different effects on lung cancer risk. High intensity occupational physical activity was associated with increased risk of lung cancer, especially nonsmall cell lung cancer, while recreational physical activity was associated with reduced risk of lung cancer, especially for nonsmall cell lung cancer, in current and former smokers, people with no history of chronic lung disease and people with normal or high BMI. Further research with a prospective design would be valuable to confirm the effect of physical activity on lung cancer.

## Acknowledgments

We thank all the staff at the Department of Thoracic Surgery, First Affiliated Hospital of Fujian Medical University. We also express our appreciation to all the patients and healthy volunteers who participated in our study.

## References

[1] Ferlay J Soerjomataram I Dikshit R Cancer incidence and mortality worldwide: sources, methods and major patterns in GLOBOCAN 2012. Int J Cancer 2015;136:E359–86. 25220842 10.1002/ijc.29210

[2] Chen WQ Zheng RS Zhang SW Report of Cancer Incidence and Mortality in China, 2012. (Chinese). Chinese Cancer 2016;25:1–8. 10.1007/s11670-012-0001-6 PMC3555260 23359628

[3] Zheng R Zeng H Zuo T Lung cancer incidence and mortality in China, 2011. Thorac Cancer 2016;7:94–9. 26816543 10.1111/1759-7714.12286 PMC4718125

[4] Bunn PA Jr Worldwide overview of the current status of lung cancer diagnosis and treatment. Arch Pathol Lab Med 2012;136:1478–81. 23194039 10.5858/arpa.2012-0295-SA

[5] Amos CI Xu W Spitz MR Is there a genetic basis for lung cancer susceptibility? Recent Results Cancer Res 1999;151:3–12. 10337715 10.1007/978-3-642-59945-3_1

[6] WHO. Cancer key facts. Available at: http://www.who.int/mediacentre/factsheets/fs297/en/ Accessed 10 Mar 2017.

[7] Garcia-Aymerich J Lange P Benet M Regular physical activity modifies smoking-related lung function decline and reduces risk of chronic obstructive pulmonary disease: a population-based cohort study. Am J Respir Crit Care Med 2007;175:458–63. 17158282 10.1164/rccm.200607-896OC

[8] Schmid D Ricci C Behrens G Does smoking influence the physical activity and lung cancer relation? A systematic review and meta-analysis. Eur J Epidemiol 2016;31:1173–90. 27502335 10.1007/s10654-016-0186-y

[9] Ho V Parent ME Pintos J Physical activity and lung cancer risk in men and women. Cancer Causes Control 2017;28:309–18. 28247218 10.1007/s10552-017-0872-4

[10] Brenner DR Yannitsos DH Farris MS Leisure-time physical activity and lung cancer risk: a systematic review and meta-analysis. Lung Cancer 2016;95:17–27. 27040847 10.1016/j.lungcan.2016.01.021

[11] Steindorf K Friedenreich C Linseisen J Physical activity and lung cancer risk in the European Prospective Investigation into Cancer and Nutrition Cohort. Int J Cancer 2006;119:2389–97. 16894558 10.1002/ijc.22125

[12] Colbert LH Hartman TJ Tangrea JA Physical activity and lung cancer risk in male smokers. Int J Cancer 2002;98:770–3. 11920649 10.1002/ijc.10156

[13] Rundle A Richie J Steindorf K Physical activity and lung cancer among non-smokers: a pilot molecular epidemiological study within EPIC. Biomarkers 2010;15:20–30. 20050820 10.3109/13547500903186452 PMC3696993

[14] Lin Y Cai L Environmental and dietary factors and lung cancer risk among Chinese women: a case-control study in southeast China. Nutr Cancer 2012;64:508–14. 22489989 10.1080/01635581.2012.668743

[15] Lin Y He F Zhang X Polymorphism rs144848 in BRCA2 may reduce lung cancer risk in women: a case-control study in southeast China. Tumori 2016;102:150–5. 26979245 10.5301/tj.5000473

[16] Zhao W Cong L [Physical activity evaluation: metabolic equivalent intensity levels and evaluation of different physical activity]. Wei Sheng Yan Jiu 2004;33:246–9. 15209020

[17] Zhang W Xiang YB Progress in epidemiological study on the relationship between physical activity and lung cancer. Acta Cancer Res 2010;37:1206–9.

[18] WHO. Global Strategy on Diet, Physical Activity and Health. Available at: http://www.who.int/dietphysicalactivity/physical_activity_intensity/en/ Accessed August 24, 2017.

[19] Wenhua Z Classification of physical activity. Health Res 2004;33:246–9.

[20] Alfano CM Klesges RC Murray DM Physical activity in relation to all-site and lung cancer incidence and mortality in current and former smokers. Cancer Epidemiol Biomarkers Prev 2004;13:2233–41. 15598785

[21] Brownson RC Chang JC Davis JR Physical activity on the job and cancer in Missouri. Am J Public Health 1991;81:639–42. 2014869 10.2105/ajph.81.5.639 PMC1405078

[22] Huang XE Hirose K Wakai K Comparison of lifestyle risk factors by family history for gastric, breast, lung and colorectal cancer. Asian Pac J Cancer Prev 2004;5:419–27. 15546249

[23] Inoue M Iso H Yamamoto S Daily total physical activity level and premature death in men and women: results from a large-scale population-based cohort study in Japan (JPHC study). Ann Epidemiol 2008;18:522–30. 18504139 10.1016/j.annepidem.2008.03.008

[24] Kubik A Zatloukal P Tomasek L Lung cancer risk among nonsmoking women in relation to diet and physical activity. Neoplasma 2004;51:136–43. 15190423

[25] Lam TK Moore SC Brinton LA Anthropometric measures and physical activity and the risk of lung cancer in never-smokers: a prospective cohort study. PLoS ONE 2013;8:e70672. 23940620 10.1371/journal.pone.0070672 PMC3734257

[26] Laukkanen JA Pukkala E Rauramaa R Cardiorespiratory fitness, lifestyle factors and cancer risk and mortality in Finnish men. Eur J Cancer 2010;46:355–63. 19683431 10.1016/j.ejca.2009.07.013

[27] Lee IM Paffenbarger RS Jr Physical activity and its relation to cancer risk: a prospective study of college alumni. Med Sci Sports Exerc 1994;26:831–7. 7934755

[28] Lee IM Sesso HD Paffenbarger RS Jr Physical activity and risk of lung cancer. Int J Epidemiol 1999;28:620–5. 10480687 10.1093/ije/28.4.620

[29] Leitzmann MF Koebnick C Abnet CC Prospective study of physical activity and lung cancer by histologic type in current, former, and never smokers. Am J Epidemiol 2009;169:542–53. 19126591 10.1093/aje/kwn371 PMC2727183

[30] Mao Y Pan S Wen SW Physical activity and the risk of lung cancer in Canada. Am J Epidemiol 2003;158:564–75. 12965882 10.1093/aje/kwg186

[31] Severson RK Nomura AM Grove JS A prospective analysis of physical activity and cancer. Am J Epidemiol 1989;130:522–9. 2763997 10.1093/oxfordjournals.aje.a115366

[32] Sinner P Folsom AR Harnack L The association of physical activity with lung cancer incidence in a cohort of older women: the Iowa Women's Health Study. Cancer Epidemiol Biomarkers Prev 2006;15:2359–63. 17164357 10.1158/1055-9965.EPI-06-0251

[33] Sprague BL Trentham-Dietz A Klein BE Physical activity, white blood cell count, and lung cancer risk in a prospective cohort study. Cancer Epidemiol Biomarkers Prev 2008;17:2714–22. 18843014 10.1158/1055-9965.EPI-08-0042 PMC2692679

[34] Thune I Lund E The influence of physical activity on lung-cancer risk: a prospective study of 81,516 men and women. Int J Cancer 1997;70:57–62. 8985091 10.1002/(sici)1097-0215(19970106)70:1<57::aid-ijc9>3.0.co;2-5

[35] Yun YH Lim MK Won YJ Dietary preference, physical activity, and cancer risk in men: National Health Insurance Corporation Study. BMC Cancer 2008;8:366–83. 19077256 10.1186/1471-2407-8-366 PMC2631012

[36] Kubik AK Zatloukal P Tomasek L Lung cancer risk among Czech women: a case-control study. Prev Med 2002;34:436–44. 11914050 10.1006/pmed.2001.1002

[37] Albanes D Blair A Taylor PR Physical activity and risk of cancer in the NHANES I population. Am J Public Health 1989;79:744–50. 2729471 10.2105/ajph.79.6.744 PMC1349635

[38] Bak H Christensen J Thomsen BL Physical activity and risk for lung cancer in a Danish cohort. Int J Cancer 2005;116:439–44. 15800940 10.1002/ijc.21085

[39] Batty GD Shipley MJ Marmot M Physical activity and cause-specific mortality in men: further evidence from the Whitehall study. Eur J Epidemiol 2001;17:863–9. 12081106 10.1023/a:1015609909969

[40] Hallmarker U James S Michaelsson K Cancer incidence in participants in a long-distance ski race (Vasaloppet, Sweden) compared to the background population. Eur J Cancer 2015;51:558–68. 25670239 10.1016/j.ejca.2014.12.009

[41] Knekt P Raitasalo R Heliovaara M Elevated lung cancer risk among persons with depressed mood. Am J Epidemiol 1996;144:1096–103. 8956621 10.1093/oxfordjournals.aje.a008887

[42] Kubik A Zatloukal P Tomasek L A case-control study of lifestyle and lung cancer associations by histological types. Neoplasma 2008;55:192–9. 18348651

[43] Land SR Liu Q Wickerham DL Cigarette smoking, physical activity, and alcohol consumption as predictors of cancer incidence among women at high risk of breast cancer in the NSABP P-1 trial. Cancer Epidemiol Biomarkers Prev 2014;23:823–32. 24569437 10.1158/1055-9965.EPI-13-1105-T PMC4011972

[44] Parent ME Rousseau MC El-Zein M Occupational and recreational physical activity during adult life and the risk of cancer among men. Cancer Epidemiol 2011;35:151–9. 21030330 10.1016/j.canep.2010.09.004

[45] Pukkala E Poskiparta M Apter D Life-long physical activity and cancer risk among Finnish female teachers. Eur J Cancer Prev 1993;2:369–76. 8401170 10.1097/00008469-199309000-00002

[46] Schnohr P Gronbaek M Petersen L Physical activity in leisure-time and risk of cancer: 14-year follow-up of 28,000 Danish men and women. Scand J Public Health 2005;33:244–9. 16087486 10.1080/14034940510005752

[47] Sormunen J Backmand HM Sarna S Lifetime physical activity and cancer incidence—a cohort study of male former elite athletes in Finland. J Sci Med Sport 2014;17:479–84. 24239090 10.1016/j.jsams.2013.10.239

[48] Steenland K Nowlin S Palu S Cancer incidence in the National Health and Nutrition Survey I. Follow-up data: diabetes, cholesterol, pulse and physical activity. Cancer Epidemiol Biomarkers Prev 1995;4:807–11. 8634649

[49] Wannamethee SG Shaper AG Walker M Physical activity and risk of cancer in middle-aged men. Br J Cancer 2001;85:1311–6. 11720466 10.1054/bjoc.2001.2096 PMC2375260

[50] Dosemeci M Hayes RB Vetter R Occupational physical activity, socioeconomic status, and risks of 15 cancer sites in Turkey. Cancer Causes Control 1993;4:313–21. 8347780 10.1007/BF00051333

[51] Guan WJ Zheng XY Chung KF Impact of air pollution on the burden of chronic respiratory diseases in China: time for urgent action. Lancet 2016;388:1939–51. 27751401 10.1016/S0140-6736(16)31597-5

[52] Lee SA Xu WH Zheng W Physical activity patterns and their correlates among Chinese men in Shanghai. Med Sci Sports Exerc 2007;39:1700–7. 17909395 10.1249/mss.0b013e3181238a52

[53] Friedenreich CM Physical activity and cancer prevention: from observational to intervention research. Cancer Epidemiol Biomarkers Prev 2001;10:287–301. 11319168

[54] Mora S Lee IM Buring JE Association of physical activity and body mass index with novel and traditional cardiovascular biomarkers in women. JAMA 2006;295:1412–9. 16551713 10.1001/jama.295.12.1412

[55] Balagopal P George D Patton N Lifestyle-only intervention attenuates the inflammatory state associated with obesity: a randomized controlled study in adolescents. J Pediatr 2005;146:342–8. 15756217 10.1016/j.jpeds.2004.11.033

[56] Kohut ML McCann DA Russell DW Aerobic exercise, but not flexibility/resistance exercise, reduces serum IL-18, CRP, and IL-6 independent of beta-blockers, BMI, and psychosocial factors in older adults. Brain Behav Immun 2006;20:201–9. 16504463 10.1016/j.bbi.2005.12.002

